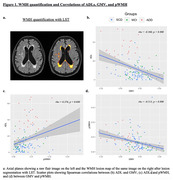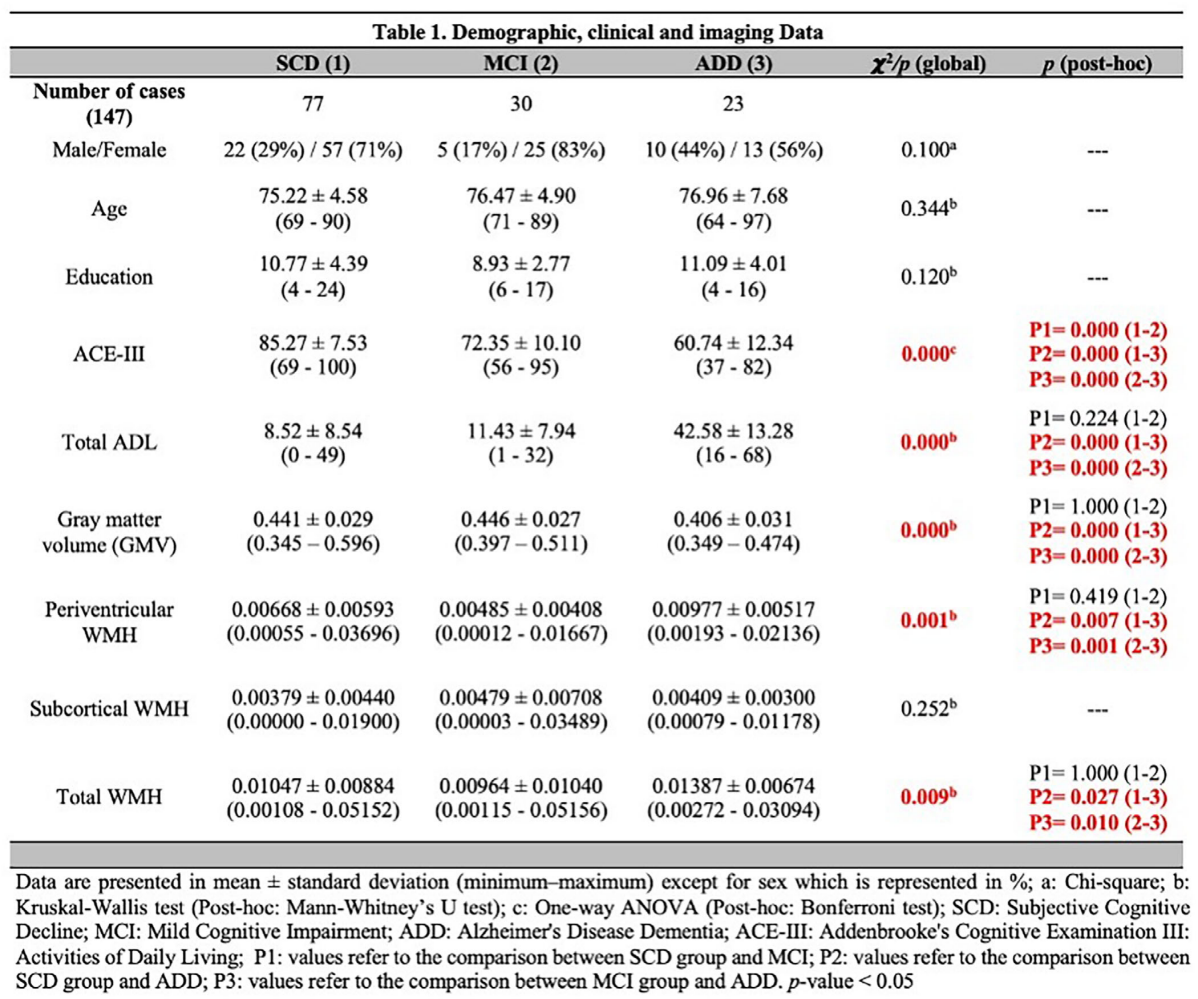# Impact of Grey Matter Volume and White Matter Hyperintensities on Functional Capacity for Activities of Daily Living: A Study in the Alzheimer’s Disease Continuum

**DOI:** 10.1002/alz.093831

**Published:** 2025-01-09

**Authors:** Cecilia Gonzalez Campo, Fernando Henriquez, Patricio Riquelme Contreras, Patricia Lillo, David Martínez‐Pernía, Daniela Thumala, Francisco Aboitiz, Andrea Slachevsky Chonchol, Rodrigo Henriquez

**Affiliations:** ^1^ CONICET, Buenos Aires Argentina; ^2^ Neuropsychology and Clinical Neuroscience Laboratory (LANNEC), Physiopathology Department ‐ ICBM, Neuroscience and East Neuroscience Departments, Faculty of Medicine, Universidad de Chile, Santiago Chile; ^3^ Geroscience Center for Brain Health and Metabolism (GERO), Santiago Chile; ^4^ Interdisciplinary Center for Neuroscience (NeuroUC) ‐ Laboratory for Cognitive and Evolutionary Neuroscience ‐ Medicine School ‐ Pontificia Universidad Católica de Chile, Santiago Chile

## Abstract

**Background:**

The Alzheimer’s disease (AD) continuum is composed of the stages of Subjective Cognitive Decline (SCD), Mild Cognitive Impairment (MCI), and Alzheimer’s Disease Dementia (ADD). The decrease in gray matter volume (GMV) secondary to cortical atrophy, commonly seen in this continuum, is related to cognitive and activities of daily living (ADL) impairment. Additionally, White Matter Hyperintensities (WMH), MRI abnormalities frequently observed in older adults and patients with dementia, are also associated with cognitive and ADL performance. This study aimed to evaluate the relationship of GMV and WMH on ADL performance across the AD continuum.

**Method:**

We conducted a cross‐sectional study of 77 SCD, 30 MCI, and 23 ADD subjects, matched for age, sex, and education. ADL was assessed using the Technology‐Activities of Daily Living Questionnaire (T‐ADLQ). Total GMV was calculated via voxel‐based morphometry analysis in SPM12. Total (tWMH), periventricular (pWMH), and subcortical (sWMH) WMH lesions were calculated with the lesion prediction algorithm (LST) and the ALVIN mask (Figure 1a). Spearman correlation analysis was used to test associations among GMV, WMH, and ADL. A linear regression model was employed to assess the relative effect of GMV and WMH on ADL scores.

**Result:**

ADD patients performed worse on ADL and showed decreased GMV and higher pWMH burden in comparison with all the other AD continuum groups (Table 1). Within the whole sample, worse ADL scores correlated significantly with decreased GMV and higher pWMH load (Figure 1b and 1c, respectively). In addition, lower GMV was correlated with greater pWMH load (Figure 1d). The linear regression model (R2 = 0.183, F = 13.890, p = 0.000) showed that only GMV had a significant impact (Beta = ‐0.338, p = 0.000), while pWMH load did not significantly influence ADL performance (Beta = 0.164, p = 0.064).

**Conclusion:**

Lower GMV and higher pWMH load are associated with worse ADL performance. These features differentially contribute to explain ADL performance across the AD continuum, with a more pronounced effect of GMV over pWMH. Widespread GM atrophy and pWMH burden are distinctive features of ADD, strongly suggesting a fundamental detrimental role of these structural brain abnormalities along the AD continuum.